# Editorial: Immune Surveillance of the HIV Reservoir: Mechanisms, Therapeutic Targeting and New Avenues for HIV Cure

**DOI:** 10.3389/fimmu.2020.00070

**Published:** 2020-01-31

**Authors:** Julia G. Prado, John Frater

**Affiliations:** ^1^IrsiCaixa AIDS Research Institute, Barcelona, Spain; ^2^Germans Trias i Pujol Research Institute, Universitat Autonoma de Barcelona, Barcelona, Spain; ^3^Nuffield Department of Medicine, University of Oxford, Oxford, United Kingdom; ^4^NIHR Biomedical Research Centre, University of Oxford, Oxford, United Kingdom

**Keywords:** HIV, reservoir, immunity, cure, CD8, BNAB, NK, immunotherapy

Thirty-eight years after the first reports of HIV infection and the start of the AIDS pandemic (1), HIV is now a manageable condition. Where there is access to highly effective antiretroviral therapy (ART), the life expectancies of those living with HIV are no different to those without, and although there is some evidence for persisting non-AIDS morbidity in a few, the majority of individuals with suppressed viraemia on ART remain well (2). In addition, through PrEP, barrier methods and the knowledge that “Undetectable Equals Untransmissable” (U=U), HIV transmission can–with the right support and infrastructure–be prevented (3).

However, despite these enormous leaps forward, there is no scalable cure for HIV. Two confirmed cases of cure following CCR5-Delta 32 stem cell transplantations and evidence of extensive drug-free viral suppression in the “post-treatment controllers” of the VISCONTI cohort provide tantalizing proof of principle that HIV can be cured or put into “virological remission” (4–6). Nevertheless, no strategy has yet been identified to replicate these cases on a large scale. In addition, it is widely hypothesized that any successful cure intervention is likely to require an effective anti-HIV immune response as part of the armory. This collection of reviews, mini-reviews and original research presents a view on some of the cellular and humoral strategies that might prove valuable.

Among the different arms of antiviral immunity, the CD8+ T cell mediated immunity has always provided one of the strongest signals of HIV control (7). Early in the epidemic it became clear that cytotoxic T cells play a major role in virological control, even inducing “elite control” status with undetectable viremia in some infected individuals (8). Whether CD8+ T cells are also important in preventing viral rebound from the reservoir of latently infected cells remains a moot point. In their review Warren et al. explore how CD8+ T cells have proven efficacy at controlling HIV viraemia, but become progressively exhausted and dysfunctional over time. ART can reverse much of this impairment (although not completely–with persistently elevated levels of immune activation on treatment), but the authors argue that other interventions that may re-focus the immune response or induce stronger *de novo* responses may be needed as part of a strategy to allow virological remission in the absence of ART. This idea is expanded by Yang et al. who explore how T cells can be engineered to be increasingly effective against the HIV reservoir, digging further into the potential of approaches such as CAR T cells, BiTEs, DARTs, and ImmTAVs, which are starting to show promise within cancer therapeutics and in animal models for HIV. In particular, their ability to non-specifically recruit T cells–including those that are not exhausted–to antigen that is expressed at very low levels could be of particular value in tackling the HIV reservoir. However, this will need to be balanced with the increased potential for off-target effects including cytotoxicity and potential autoimmune complications.

Clutton and Jones reinforce this message that potential promising interventions may come with unwanted effects, where they explore how the act of inducing the “kick” as part of a “kick and kill” strategy, may have negative connotations for T cell function, thereby undermining the “kill” component. They focus on histone deacetylase inhibitors (HDACi) and protein kinase C agonists (PKCa), and explore whether–even if viral antigen presentation is enhanced–they can simultaneously impact T cell viability, proliferative potential, degranulation, and killing.

This is explored further by Ruiz et al., where they developed a novel “Kick and kill assay” to determine the effectiveness of CTL following LRA treatments, and show that although there is evidence of killing there may be multiple inhibitory factors to be overcome. They find evidence for increased killing of reactivated cells (proportional to the degree of reactivation), but with significant inter-individual variation, partly explained by CD8+ T cellular dysfunction associated with the co-expression of inhibitory receptors including PD-1, Tim-3, and LAG-3.

However, these negative effects of immune checkpoint markers may be turned on their heads according to Boyer and Palmer, where they describe how although molecules such as PD-1 are markers of dysfunction on CD8+ T cells, they have an additional role on CD4+ T cells as markers of enrichment of HIV-latent infection. As such, they argue that monoclonal antibodies targeted against these molecules (e.g., nivolumab vs. PD-1 and ipilimumab vs. CTLA-4) might effectively reverse latency, as well as improving CTL functionality, as they do in cancer therapeutics. However, the clear message that toxicities and side-effects of Immune Checkpoint (IC) molecules may undermine any positive effects is not lost on the authors, and it is clear that future HIV treatment strategies need to be safe as well as effective.

In the context of finding a biomarker for the HIV reservoir, whereas IC molecules show evidence for enrichment, it has been suggested that the FC gamma receptor CD32 may be a more specific marker (9). Due to the importance and implications of this finding, many investigators turned to CD32, including Martin et al.. Although they were able to find evidence of CD32 expression in analysis of cells that contained HIV DNA, there was no correlation with total or integrated HIV DNA levels (or time to rebound on stopping ART), consistent with subsequent findings that CD32 may in fact be a marker of T cell—B cell doublets that have survived flow cytometry gating (10), but this remains a contentious area.

Also considered as a biomarker for the reservoir is CD2, and this is explored by Tomalka et al. who present a study exploring the role of Alecfacept. Although historically, CD8+ T cells have been the focus of most attention, there is increasing evidence from cohorts like VISCONTI that innate immunity and NK cells may be critical effectors in future cure strategies. Tomalka et al. present an *in vitro* data using an anti-CD2 monoclonal antibody, alefacept, to induce natural killer (NK) cell-driven antibody-dependent cell-mediated cytotoxicity (ADCC). Specifically targeting CD45RA- CD4+ memory T cells, they present evidence to show that killing can be induced and that this can reduce HIV DNA levels in patient samples *ex vivo*. Whilst very early steps toward clinical application, studies such as these provide proof-of-principle that alternative immunological effectors–in this case NK cells–may have a potential role in cure strategies.

This message underpins the current enthusiasm for the role of broadly neutralizing antibodies (bNAbs) following exciting studies in macaques and humans showing evidence of virological suppression and some suggestion of a post-therapy “vaccinal effect” which may confer longer-term protection (11–13). This topical area is reviewed by Carrillo et al.. They provide a detailed and comprehensive review of the technologies that have allowed the frameshift toward effective bNAbs, and how these new agents show antiviral activity initially in animal models and now in human clinical trials. The potential for bNAbs both on their own and in conjunction with other interventions could have an enormous impact on both future treatment and prevention strategies.

Overall, the aim of this collection was to provide an overview of how the immune system remains a key player in future HIV therapeutics. Our current best treatment strategies are highly effective and have revolutionized the course of HIV infection. However, there is always room for improvement, and these papers explore how new agents and approaches might eventually lead to long-acting sustained responses, ideally in the absence of side-effects. These long-lasting immune responses will lead the continuous immune surveillance of infected cells in the body, allowing the control or elimination of the HIV reservoir.

It is an exciting time to consider how the leaps forward in treatments for cancer and autoimmune conditions, can also help people living with HIV. It is important not to become complacent and to always strive for better. We wish to convey our appreciation to all the authors who have participated in this Research Topic and the reviewers for their insightful comments.

## Author Contributions

All authors listed have made a substantial, direct and intellectual contribution to the work, and approved it for publication.

### Conflict of Interest

The authors declare that the research was conducted in the absence of any commercial or financial relationships that could be construed as a potential conflict of interest.
